# Debates in allergy medicine: baked milk and egg ingestion accelerates resolution of milk and egg allergy

**DOI:** 10.1186/s40413-015-0089-5

**Published:** 2016-01-26

**Authors:** Stephanie A. Leonard

**Affiliations:** Division of Pediatric Allergy & Immunology, University of California, San Diego, Rady Children’s Hospital San Diego, 3020 Children’s Way, MC 5114, San Diego, CA 92123 USA

**Keywords:** Baked egg, Baked milk, Egg allergy, Milk allergy

## Abstract

Cow’s milk and hen’s egg are ubiquitous in diets around the world and can be important sources of protein in young children. Unfortunately, milk and egg allergies are also some of the most common food allergies in childhood. Less allergenic forms of milk and egg due to heating and interactions with a food matrix, as in baked goods, are tolerated by a majority of milk- and egg-allergic patients. Adding baked milk and egg into the diets of milk- and egg-allergic children can broaden diets, increase nutrition, and improve quality of life. Most important, regular ingestion of baked milk and egg can help children outgrow their allergies to milk and egg. This article will review our current understanding of baked milk and egg tolerance and outline how these baked forms accelerates tolerance to regular milk and egg.

## Background

Milk and egg allergies are among the most common food allergies in children. Milk allergy affects 1.4–3.8 % of young children in the United States and Europe, with a population based study in Israel reporting IgE-mediated milk allergy in 0.5 % of infants [1–5]. Egg allergy affects 1–2.6 % of young children in the United States and Europe, with an Australian study reporting raw egg allergy in 8.9 % of infants [1–3, 6, 7]. Up to 80 % of children may outgrow milk and egg allergy, however, while it was previously thought that tolerance developed in a majority by school age, recent data suggests that it is taking longer to outgrow these allergies [8–10]. In addition, the prevalence of food allergy continues to increase and currently there is no cure [11]. Oral immunotherapy to mitigate food allergy is not available in routine clinical practice in most countries. The primary management of food allergy remains strict avoidance of food allergens and prompt treatment of accidental ingestions and reactions. Strategies that could help children outgrow milk and egg allergies would be invaluable.

Milk and egg are common food ingredients, as well as important sources of protein in young children. The ability to add safer, less allergenic forms of milk and egg protein, such as in baked goods, into the diets of milk- and egg-allergic children has several benefits. First, it broadens the diet and decreases the risk of nutritional deficiencies or feeding issues in young children. Second, it improves quality of life by increasing dietary options and allowing children to feel more included in social situations, such as birthday parties [12]. And lastly, it can decrease the time to development of tolerance to regular milk and egg. This article will show that baked milk and egg is well tolerated and can help children outgrow their milk or egg allergy.

### Food processing

Food processing alters proteins and can make them more or less allergenic. For example, conformational epitopes can be denatured by heat, while linear epitopes are likely to stay intact [13]. In addition, heat can affect individual protein components differently. Casein in milk and ovomucoid in egg are heat stable, while α-lactalbumin and β-lactoglobulin in milk and ovalbumin in egg are heat labile [14]. Studies have shown that milk- and egg-allergic patients with predominant IgE binding to linear epitopes of casein and ovomucoid are more likely to have persistent milk and egg allergy [15, 16]. Using serum from milk- and egg-allergic patients, Bloom et al. demonstrated that heating decreased IgE binding to milk and egg proteins, and that all baked milk-reactive patients retained IgE binding to casein, regardless of heating time [14].

Besides alterations due to heating, the interaction of milk and egg proteins with a food matrix, such as wheat, has also been shown to decrease IgE recognition. Bloom et al. and Shin et al. used SDS-PAGE to compare IgE binding of baked milk and egg vs. regular milk and egg heated under the same conditions and found that allergenicity decreased in the presence of wheat, even to heat stable casein and ovomucoid proteins [14, 17]. The authors of Bloom et al. hypothesized that milk and egg proteins form complexes with wheat that make the proteins less available to the immune system and decreasing their effective allergenicity.

Studies have shown that subjects can tolerate a larger amount of milk or egg protein in baked goods compared to regular milk or egg alone. Turner et al. noted that tolerance of baked egg was not simply due to smaller amounts of egg ingested since a number of patients tolerated over 1 gram of baked egg protein in a muffin, yet remained reactive to under 300 mg of regular (unbaked) egg protein [18]. Likewise, Lemon-Mule et al. reported subjects who tolerated 2.2 g of baked egg protein during a muffin challenge, yet reacted to a median dose of 1.5 g of regular egg protein, with 75 % reacting to less than 2.2 g [19]. Nowak et al. had similar findings in baked milk, reporting subjects who tolerated 1.3 g of baked milk protein during a muffin challenge yet reacted to a median dose of 0.4 g regular (unbaked) milk protein [20]. This supports the theory that IgE recognition of milk and egg proteins in baked goods is altered and less allergenic.

### Tolerability of baked milk and egg

Studies show that a majority of milk- and egg-allergic children can tolerate baked milk or egg. In cohort studies, between 69 and 83 % of milk-allergic subjects tolerated baked milk (Table 1) [20–23]. Similarly, between 63 and 84 % of egg-allergic subjects tolerated baked egg (Table 2) [18, 19, 24–30].

**Table 1:** Tolerance of baked milk and resolution of milk allergy

	Type of study	BM tolerant	Resolution of milk allergy
Wood et al. [39] (*n* = 155)	Observational without OFC	21 % by 5 years	Relative hazard after 5 years:
			4.1 in those who reported BM tolerance
			0.28 in those who reported BM reactivity
Nowak-Wegrzyn et al. [20] (*n* = 91) and Kim et al. [32] (*n* = 88; follow-on study)	Prospective with OFC	75 % initially, 80 % by end of follow-on study	59 % of those ingesting BM (followed for median 37 months)
			22 % of control group (followed for median 40 months)
Caubet et al. [21] (*n* = 121)*	Prospective with OFC	69 %	N/A
Bartnikas et al. [22] (*n* = 35)	Retrospective with OFC	83 %	N/A
Mehr et al. [23] (*n* = 70)	Prospective with OFC	73 %	N/A

*OFC* oral food challenge, *BM* baked milk

*only second cohort; first cohort include subjects from Nowak et al

**Table 2:** Tolerance of baked egg and resolution of egg allergy

	Type of study	BE tolerant	Resolution of egg allergy
Sicherer et al. [40] (*n* = 113)	Observational without OFC	38 % by 6 years	After 6 years:
			71 % in those who reported BE tolerance
			45 % in those not ingesting BE
			57 % in those who reported BE reactivity
Des Roches et al. [30]	Prospective with OFC	73 %	
Lemon-Mule et al. [19] (*n* = 91) and Leonard et al. [31] (*n* = 79; follow-on study)	Prospective with OFC	70 % initially,	53 % of those ingesting BE (followed for median 37.8 months)
		89 % by end of follow-on study	28 % of control group (followed for median 67.3 months)
Clark et al. [24] (*n* = 95)	Longitudinal with OFC	66 %	N/A
Lieberman et al. [25] (*n* = 100)	Retrospective with OFC	66 %	N/A
Cortot et al. [26] (*n* = 52)	Retrospective with OFC	83 %	N/A
Turner et al. [18] (*n* = 236)	Prospective with OFC	64 %	N/A
Tan et al. [27] (*n* = 143)	Prospective with OFC	63 %	N/A
Bartnikas et al. [28] (*n* = 169)	Retrospective with OFC	84 %	N/A
Turner et al. [29] (*n* = 186)	Retrospective with OFC	66 %	N/A
Peters et al. [37] (*n* = 158)	Prospective with OFC or by report	80 % by OFC	By age 2 years of age:
		72 % by report	49 % of those ingesting BE after OFC at 1 year
			74 % of those ingesting BE by report at 1 year
			13 % of those with positive BE OFC at 1 year

*OFC* oral food challenge, *BE* baked egg

In two prospective cohort studies at a large tertiary care center, Nowak et al. and Lemon-Mule et al. demonstrated that the ingestion of baked milk and egg, respectively, was well tolerated [19, 20]. There was no difference in weight, height and body mass index for age and z score at baseline compared to after 12 months of ingesting baked milk or egg. Intestinal permeability in those ingesting baked milk or egg was not significantly affected and other atopic diseases did not worsen. There were no reported immediate reactions to baked milk or egg prepared per study protocol at home after passing a baked milk or egg oral food challenge (OFC). One baked milk-tolerant subject subsequently reported oral pruritus to insufficiently baked milk items (homemade bread and waffles), but tolerated a repeat baked milk OFC and resumed sufficiently baked milk products at home [20]. One baked egg-tolerant subject developed delayed vomiting and diarrhea after accidental ingestion of regular egg consistent with food protein-induced enterocolitis syndrome, and subsequently returned to avoiding all forms of egg [31]. No subject ingesting baked milk or egg developed eosinophilic esophagitis determined to be due to milk or egg [32].

Symptoms to baked milk and egg at home after passing a baked milk or egg OFC have been reported in other studies, however it is not possible to verify whether the foods were properly baked. In Bartnikas et al., three subjects who passed a baked milk OFC developed symptoms after ingesting baked milk at home; none returned for a repeat baked milk food OFC [22]. In addition, one of these subjects, who reported oral pruritus after baked milk ingestion at home, also developed oral pruritus during the baked milk OFC that they were ultimately determined to have passed. In Mehr et al., three children who passed a baked milk OFC reported mild symptoms at home (pruritus, abdominal pain and eczema) one week later and were assumed to be following the same recipe used in the OFC [23]. In Turner et al., two subjects who tolerated the baked egg OFC developed abdominal symptoms upon ingestion at home one week later [18]. Without a repeat baked milk or egg OFC, it is difficult to know if baked milk or egg products at home were sufficiently baked or if these subjects subsequently developed sensitivity. Baked milk and egg recipes and recommendations on how to introduce baked milk and egg into the diets of children who are tolerant, are available in a review by Leonard et al. [33].

Adherence to baked milk and egg diets appears to be good. In a study by Lee et al. follow-up on milk- and egg-allergic subjects who passed a baked milk or egg (muffin) challenge and were encouraged to ingest muffins on an at least weekly basis was studied by survey [34]. Of 98 respondents, 72 % reported that they were still ingesting muffins at a median time of 12 months since their challenge, with 68 % ingesting some form of baked milk or egg on an at least weekly basis, and only 10 % not ingesting any form of baked milk or egg. About 68 % of parents appeared motivated to include baked milk and egg in the diet based on the possibility that it may help their child outgrow their milk or egg allergy. A majority of parents also reported that performing the muffin challenge helped to relieve concerns about their child’s allergy (82 %) and helped with dietary management (77 %).

Of those who discontinued ingesting muffins in Lee et al., less than half (12 of 27; 44 %) stopped due to symptoms [34]. Other reasons for stopping were not delineated. History, time since, and severity of prior reactions to milk or egg and baseline characteristics were not predictive of which subjects discontinued. Symptoms were reported to be predominantly mild and gastrointestinal, and interestingly, 19 subjects who experienced symptoms continued to ingest muffins. The authors did not address whether symptoms resolved over time while muffins were still being ingested, or whether these subjects were more or less likely to develop tolerance to regular milk or egg.

### Predictability of baked milk and egg tolerance

While established skin prick test (SPT) wheal and specific IgE level cut-offs for regular milk and egg reactivity have been clinical useful, data is inconsistent for predicting baked milk and egg reactivity (Tables 3 and 4). Variability is likely due to the heterogeneity of baked milk and egg protocols as well as study population characteristics. Calvani et al. performed a systemic review of the ability of egg-specific SPT and IgE levels to predict reactivity to baked egg, and concluded that the studies, which proposed different cut-offs, were at high risk for bias [35]. In many studies, the larger the SPT wheal and the higher the specific IgE level to cow’s milk or egg white, the less likely the patient would tolerate baked milk or egg [19–22, 25, 27–29]. However, even some patients with large SPT wheals and high specific IgE levels to cow’s milk and egg white tolerated baked milk and egg. In Turner et al., while baked egg- tolerant subjects were more likely to have lower SPT wheals to both egg extract (EE) and raw egg (RE) and lower EE/RE ratio, these measurements showed poor predictability of challenge outcomes [29]. And lastly, Lieberman et al. reported that both baked egg tolerant and reactive subjects had the identical median SPT wheal of 7 mm [25]. These data indicate that while there are trends in SPT and IgE levels, reliable cut-offs for predicting baked milk and egg reactivity are still needed.

**Table 3:** Predictability of Baked Milk Tolerance by Testing

	SPT wheal	Specific IgE (kU_A_/L)	Suggested cut-off for OFCs
Nowak-Wegrzyn et al. [20]	CM < 5 mm = 100 % NPV [poor specificity]	CM IgE ≥35 = >50 % PPV	CM IgE 5.0 kU/L
	CM 15 mm = 50 % PPV		
Caubet et al. [21]	N/A	CM IgE 24.5 = 69 % PPV [poor sensitivity]	Casein IgE 5 kU_A_/L
		Casein IgE 20.2 = 69 % PPV [poor sensitivity]	CM IgE 10 kU_A_/L
		Casein IgE 4.95 = 89 % NPV	
Bartnikas et al. [22]	CM <7 mm = 100 % NPV	CM IgE >20.6 = 100 % PPV	N/A
	Casein >15 mm = 100 % PPV	Casein IgE >10.3 = 100 % PPV	
		Casein IgE 0.9 = 90 % NPV [poor sensitivity and specificity]	

*SPT* skin prick test, *CM* cow’s milk, *PPV* positive predictive value, *NPV* negative predictive value, *OFC* oral food challenge

**Table 4:** Predictability of Baked Egg Tolerance by Testing

	SPT Wheal	Specific IgE (kU_A_/L)	Suggested cut-offs for OFCs
Lemon-Mule et al. [19]	EW 15 mm = 60 % PPV	EW IgE 75 = >50 % PPV	N/A
		OM IgE 50 = 90 % PPV	
Ando et al. [41]	N/A	EW IgE 30.7 = 84 % PPV [moderate sensitivity]	EW IgE 7.38 kU_A_/L
		OM IgE 10.8 = 88 % PPV [moderate sensitivity]	OM IgE 4.40 kU_A_/L
		OM IgE 1.16 = 97 % NPV [moderate specificity]	
Cortot et al. [26]	EW < 10 mm = 100 % NPV	EW IgE not significant	N/A
Lieberman et al. [25]	EW not significant	EW IgE 10 = 60 % PPV [poor sensitivity]	N/A
Caubet et al. [42]	N/A	EW IgE 26.2 = 43 % PPV [poor sensitivity]	EW IgE 2.6 kU_A_/L
		EW 0.78 = 97 % NPV [poor sensitivity]	OM IgE 3.3 kU_A_/L
		OM IgE 12.8 = 64 % PPV [poor sensitivity]	
Tan et al. [27]	OM ≥ 11 mm = 100 % PPV [poor sensitivity]		N/A
	Muffin <2 mm = 88 % NPV [poor specificity]		
Bartnikas et al. [28]	EW <3 mm = 100 % NPV	EW IgE 9.65 = 59 % PPV [poor sensitivity]	OM IgE 0.35 kU_A_/L
	EW < 11 mm = >90 %NPV [moderate sensitivity and specificity]	OM IgE 3.38 = 42 % PPV [poor sensitivity]	EW IgE 6.00 kU_A_/L
			EW SPT 11 mm
Turner et al. [29]	EW ≥ 12 mm = LR 9.1 [poor sensitivity]		N/A
	Raw egg ≥25 mm = LR 5.8 [very poor sensitivity]		
Peters et al. [43]	EW > 11 mm = 82 % PPV		N/A

*SPT* skin prick test, *EW* egg white, *OM* ovomucoid, *PPV* positive predictive value, *NPV* negative predictive value, *LR* likelihood ratio, *OFC* oral food challenge

The argument could be made that diagnostic testing using baked forms would be more reflective of reactivity to baked milk or egg. However, Mehr et al. found that SPT to baked milk muffin slurry did not predict reactivity to baked milk challenge [23]. Tan et al. reported that a baked egg muffin SPT < 2 mm had a 88 % negative predictive value (NPV) with 96 % sensitivity but only 17 % specificity [27].

It has been suggested that casein- and ovomucoid-specific diagnostic testing may be more useful in predicting baked milk or egg reactivity since these proteins are less affected by heating. Several studies have reported casein- and ovomucoid-specific IgE levels with high positive predictive values (PPV) and NPVs, and some have suggested cut-offs (Tables 3 and 4). Bartnikas et al., however, did not find that ovomucoid-specific IgE levels had better predictability compared with egg white-specific IgE levels [28]. Larger cohorts and a standardized protocol for baked milk and egg may be needed to establish cut-offs.

### Safety of baked milk and egg oral food challenges

Due to the inconsistencies in diagnostic testing for predicting baked milk and egg reactivity, OFC remains the most useful method for determining tolerability. Since a majority of milk- and egg-allergic children tolerate baked forms, there is debate over whether baked milk and egg can be introduced at home or if a physician-supervised OFC should be performed first. Some studies have reported mild symptoms or no use of epinephrine during baked milk and egg reactions [22, 24], while other studies have described significant reactions, including anaphylaxis. Reactivity to baked milk and egg during OFCs in Nowak et al. and Lemon-Mule et al. ranged from mild to severe with 8 out of 23 (35 %) baked milk-reactive subjects and 5 out of 27 (18.5 %) baked egg-reactive subjects receiving epinephrine [19, 20]. In other baked egg studies, between 9.4 and 22 % of baked egg reactors developed anaphylaxis, and 5.8–13 % were treated with epinephrine [18, 23, 25, 26, 28]. In general, due to the potential risk of anaphylaxis and need for epinephrine, it is suggested that physician-supervised OFCs are performed before adding baked milk or egg into the diets of milk- and egg-allergic patients, if it cannot be established that they are regularly tolerating baked milk or egg at home already [36].

### Does tolerance to baked milk and egg simply represent a milder phenotype?

It has been suggested that tolerance of baked milk and egg may simply represent a transient milk and egg allergy and thus constitute a prognostic marker, however data are not consistent. In one prospective, population-based cohort study, subjects who were baked egg reactive were 5 times more likely to have persistent egg allergy compared to subjects who were baked egg tolerant based on OFC or parental report (*P* < .001) [37]. In Nowak et al., tolerance to baked milk appeared to represent a milder phenotype in that none of baked milk tolerant subjects received epinephrine during regular milk reactions, while 35 % of baked milk reactive subjects received epinephrine for anaphylaxis during baked milk reactions [20]. This was not seen for baked egg in a similar population. Lemon et al. reported that tolerance to baked egg did not appear to represent a milder phenotype since 18.5 % of baked egg reactive and 23 % of regular egg reactive subjects received epinephrine for anaphylaxis during reactions [19]. In a different prospective study on the safety of baked egg challenges, Turner et al. reported that only 25 % of subjects who developed anaphylaxis to baked egg challenges had a prior history of anaphylaxis to regular egg, while 50 % had a non-anaphylactic reaction and 25 % had no history of previous reaction to egg [18]. In addition, 30 % of the cohort’s 114 children classified as having “mild egg allergy” (defined as non-asthmatics with a history of skin symptoms only on significant exposure to egg) reacted to baked egg; four of them developed anaphylaxis and two were treated with epinephrine.

Conversely, one could make the argument that since diagnostic testing does not appear to correlate as well with baked milk and egg reactivity as it does with regular milk and egg reactivity, that tolerance of baked milk and egg does not simply represent a milder, transient form of milk or egg allergy. If it did, then only patients with small SPT wheals or low IgE levels to cow's milk and egg white would be expected to tolerate baked milk or egg.

### Resolution of milk and egg allergy

Regular ingestion of baked milk and egg appears to play an important role in accelerating the development of tolerance to regular milk and egg. In Peters et al., among infants who were baked egg tolerant by 1 year of age, those that ingested baked egg frequently (≥5 per month) were 3 times more likely to outgrow their egg allergy than those who ingested baked egg infrequently or strictly avoided (*P* = .009) [37]. Leonard et al. demonstrated that initially baked egg-tolerant subjects were 12.2 times more likely to develop tolerance to regular egg than those who were initially baked egg-reactive (*P* < .001) [31]. Subjects who were regularly ingesting baked egg were 14.6 times more likely to develop tolerance to regular egg vs. a comparison group (*P* < .0001). Similarly, Kim et al. demonstrated that initially baked milk-tolerant subjects were 28 times more likely to develop tolerance to regular milk than those who were initially baked milk-reactive (*P* < .001) [32]. Subjects who were regularly ingesting baked milk were 16 times more likely to develop tolerance to regular milk vs. a comparison group (*P* < .001). The comparison groups in Leonard et al. and Kim et al. were matched clinic patients collected retrospectively who represented “standard of care”, or how milk and egg allergy was typically managed at that time.

In these two studies, immunologic parameters showed a similar pattern to those found during subcutaneous immunotherapy and in those who develop natural tolerance to foods. In the baked milk follow-up study, casein- and β-lactoglobulin-specific IgE levels decreased significantly and median casein-specific IgG4 levels increased significantly in subjects ingesting baked milk compared to those strictly avoiding [32]. Similarly, in the baked egg follow-up study, egg white-, ovalbumin- and ovomucoid-specific IgE levels decreased significantly and ovalbumin- and ovomucoid-specific IgG4 levels increased significantly from baseline in those ingesting baked egg compared to those strictly avoiding [31]. This supports the theory that baked milk and egg are immunologically active and can act as a treatment to help children outgrow their milk and egg allergy.

A mouse model of food allergy has also provided supporting evidence that baked egg can function as a treatment for egg allergy, in the form of oral immunotherapy (OIT). In this study, extensively heated ovomucoid, which did not trigger anaphylaxis in ovomucoid-sensitized mice, was as efficacious as unheated ovomucoid in desensitizing mice [38]. In other words, extensively-heated ovomucoid provided a safer form of OIT. It is possible that baked milk and egg in the diets of milk- and egg-allergic children may act as a safer form of OIT.

A proactive approach of including baked milk and egg in the diet can be beneficial to milk- and egg-allergic children. Reported rates of baked milk or egg ingestion are lower in observed cohorts where baked forms are not actively endorsed. In an observational study on milk allergy, only 21 % (32/155) of milk-allergic infants were tolerating some baked milk products after 5 years by parental report, and 7 reported baked milk reactivity [39]. In an observational study on egg allergy, only 38 % (43/113) of egg-allergic infants were tolerating some baked egg products after 6 years by parental report, and 4 reported baked egg reactivity [40]. In these studies, tolerance to baked milk and egg was not clinically assessed and reported tolerance levels were much lower than in studies actively challenging and introducing patients to baked milk and egg. If ingestion of baked milk and egg is viewed as a way to shorten the time to regular milk and egg tolerance, it is likely that clinical assessments for such tolerance will become more frequent.

## Conclusion

Inclusion of baked milk and egg into the diets of milk- and egg-allergic patients appears to be well tolerated and beneficial as a potential form of immunotherapy to help children outgrow their milk and egg allergy. It is unlikely that tolerance of baked milk and egg simply represents a milder or transient form of milk or egg allergy, because baseline reactivity to regular milk and egg and diagnostic testing have had limited ability to predict baked milk or egg tolerance. Even patients with moderate testing or a history of anaphylaxis to milk and egg have been able to tolerate baked milk and egg. Studies on proactive ingestion of baked milk and egg and associated changes in immunologic parameters support baked milk and egg as an active treatment for food allergy. Regular evaluation and testing is recommended to evaluate for baked milk and egg tolerance and for the development of tolerance to regular milk and egg. Due to the risk of anaphylaxis to baked milk and egg, physician-supervised OFCs are recommended to determine tolerance to baked milk and egg.

## Abbreviations

NPV: negative predictive value
OFC: oral food challenge
PPV: positive predictive value
SPT: skin prick test
